# Mapping the pathways to health sciences librarianship: reflections and future implications from an immersion session

**DOI:** 10.5195/jmla.2023.1645

**Published:** 2023-10-02

**Authors:** Gregory Laynor, Natalie Tagge, Juliana Magro, Megan De Armond, Renée A. Rau, Emily Vardell

**Affiliations:** 1 gregory.laynor@nyulangone.org, Assistant Curator & Systematic Review Librarian, NYU Health Sciences Library, NYU Grossman School of Medicine, New York, NY.; 2 ntagge@ucsd.edu, Assistant Program Director, Research Advisory Services, UC San Diego Library, University of California, La Jolla, CA.; 3 juliana.magro@nyulangone.org, Assistant Curator & Education and Research Librarian, NYU Health Sciences Library, NYU Grossman School of Medicine, New York, NY.; 4 mde_armo@touro.edu, Associate Professor & Systematic Review Librarian, Jay Sexter Library, Touro University Nevada, Henderson, NV.; 5 renee.rau@usc.edu, Information Services Librarian, Norris Medical Library, University of Southern California, Los Angeles, CA.; 6 evardell@emporia.edu, Associate Professor, School of Library and Information Management, Emporia State University, Emporia, KS.

**Keywords:** Health sciences librarianship, mentoring, Career Development, Library Staffing, Training, Continuing Education, Internships, LIS programs, self-teaching, Diversity and Inclusion in Libraries

## Abstract

**Objective:**

Many health sciences librarians enter the profession without specific health sciences training. Some LIS programs have health sciences courses or tracks, but health sciences training within an LIS program is only one path to entering health sciences librarianship. To develop a map of pathways into health sciences librarianship, an immersion session at the Medical Library Association conference in 2022 asked health sciences librarians to share how they entered the profession.

**Methods:**

The immersion session was structured in three parts: facilitator introductions, small group discussions, and a whole group summary discussion. Guided by questions from the facilitators, small groups discussed what pathways currently exist, how to promote existing pathways, what new pathways should be created, and how to develop and promote pathways that make the profession more equitable, diverse, and inclusive.

**Results:**

Through in-the-moment thematic analysis of the small group discussions, the following emerged as key pathways: library school education; internships and practica; the Library and Information Science (LIS) pipeline; on-thejob training; mentoring; self-teaching/hands-on learning; and continuing education. Themes of equity, diversity, and inclusion arose throughout the session, especially in the concluding whole group discussion.

**Conclusion:**

Small group discussions in a conference immersion session showed the value of community building in a profession that has multiple pathways for entrance, highlighting the importance of unearthing hidden knowledge about avenues for exploring and enhancing career pathways. The article seeks to address barriers to entry into the profession and adds to the literature on strengthening the field of health sciences librarianship.

## INTRODUCTION

There are multiple pathways to health sciences librarianship. Some health sciences librarians begin their careers by way of a health sciences track in a Library and Information Science (LIS) graduate program, but this is not the typical path. Meanwhile, there are concerns that general training in librarianship does not develop the specific competencies of health sciences librarianship, such as familiarity with evidence-based medicine [1]. However, as Bartley, Simuel, and Williams have written, there are often transferable skills from previous work experiences outside of health sciences libraries that librarians new to the health sciences bring to the health sciences environment [2]. Responding to the need for more introductory training in health sciences librarianship for librarians with other backgrounds, two librarians who started careers in health sciences librarianship without prior health sciences training developed an online course and mentoring program introducing health sciences librarianship as a career path [3]. Reflection on the course and mentoring program led to a proposal for a Medical Library Association conference immersion session focusing on what pathways to health sciences librarianship exist, how pathways can be better promoted, and what new pathways should be developed.

At the MLA ‘22 conference in New Orleans; the Medical Library Education Caucus sponsored the “Developing Pathways to Health Sciences Librarianship” immersion session. The session planning team, the authors of this article, came together from a variety of perspectives including health sciences librarians who started as academic librarians, health sciences librarians with backgrounds in the arts and humanities, and an LIS faculty member who teaches courses in health sciences librarianship. The immersion session was an opportunity for health sciences librarians to describe their pathways to health sciences librarianship. The session welcomed any participants who wanted to contribute to developing and promoting existing and new opportunities for introductory education and mentoring in health sciences librarianship. The session also encouraged participation from health sciences library administrators tasked with recruiting and leading search committees or supporting librarian professional development.

We discuss in this article the themes and trends that emerged as immersion session participants shared their experiences and ideas. We hope that this article advances the conversation about how to develop and promote pathways to health sciences librarianship, especially in ways that make the profession more equitable, diverse, and inclusive.

## METHODS

The immersion session was divided into three components: facilitator introductions, small group discussions, and a summary discussion with the whole group. The small group discussions were guided by five questions introduced by the facilitators:

1. What methods have you used to develop the skills needed to work as a health sciences librarian?
2. What existing opportunities for introductory health sciences librarianship training and mentoring are you aware of?
3. How might these existing opportunities be better promoted so as to increase equity, diversity, and inclusion in health sciences librarianship?
4. What new introductory health sciences librarianship education or mentoring programs are needed in order to make health sciences librarianship more equitable, diverse, and inclusive?
5. Can you envision how you may want to become involved in existing and/or new health sciences librarianship education or mentoring programs?

The authors documented thematic analysis of the discussions but did not collect individually identifiable or sensitive information from discussion participants. The first author completed and signed a “Self-Certification Form for Determining Whether Your Proposed Activity is Research Involving Human Subjects” self-certification form provided by their institution's Institutional Review Board (IRB). Based on the criteria of the self-certification form, this project did not fall under the purview of IRB review since the activity was not a systematic investigation for research but rather a group discussion with no individually identifiable information collected. The session began with a slide stating the intent to collect and publish thematic analysis of the session's discussions. Before small group discussions began, facilitators explained how they would take notes of discussions contributions, without collecting participant names, and would analyze the group's discussion for themes. Facilitators specified that if anyone did not want a discussion contribution to be noted, they were welcome to request that the discussion facilitator not report it. Although an exact count of the number of participants was not recorded, there were three tables for small group discussions, each with approximately 10-15 participants including a facilitator, totaling approximately 30-45 participants in the session.

**Figure 1:**
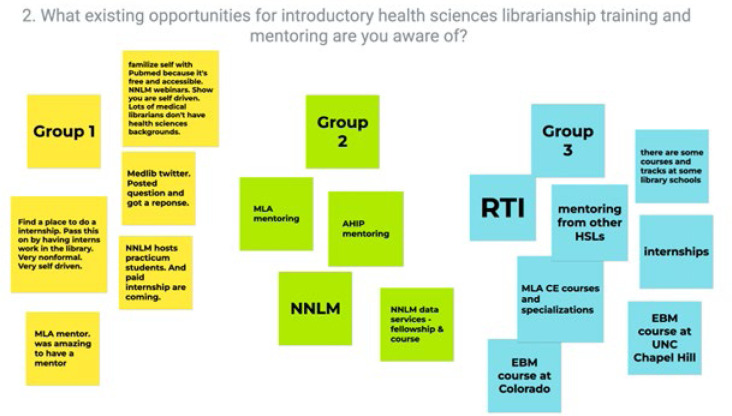
Jamboard frame representing question number 2

Facilitators aimed to analyze themes from all small group discussions in real-time and then to share with the full group a map of themes that had emerged across the small groups. Accomplishing this required technology that could simultaneously collect and organize ideas generated by multiple small groups. To aggregate contributions from the small group discussions, the facilitators decided to use Jamboard [4]. Jamboard is a digital, interactive whiteboard developed by Google, freely available to anyone with a Google account. The team created one Jamboard with five slides, corresponding to the five discussion questions. Each discussion table had one facilitator equipped with a computer. Additionally, participants had access to the Jamboard link, and could enter their responses and ideas themselves if desired. For those unfamiliar with Google Jamboard, instructions and a demo sandbox were available, all within the same Jamboard. Figure 1 below illustrates one Jamboard frame, with answers from the participants. Each table entered their answers using a different sticky note color.

While the small groups explored each question and filled out the Jamboard at their tables, three additional facilitators at the front of the room analyzed the themes being generated in real-time and used a second software tool called Miro to map the themes. Like Jamboard, Miro is also an online, collaborative whiteboard [5]. Miro has the advantage of a built-in mind mapping template, allowing for the speed that facilitators needed to synthesize contributions from multiple simultaneous discussions. In addition, Miro has a canvas that can be expanded in all directions, while Jamboard's screen is set up as a slide that limits the amount of information that can be displayed at once. Miro also allows for multiple color settings, unlike Jamboard, which favors large displays of multiple categories. One disadvantage is that Miro is not free; however, it offers a free tier with three boards. Some capabilities are limited in the free version; for example, it only exports lower resolution images. One further drawback is that users need to create accounts to access the boards. Nonetheless, the free version of Miro met the needs of this session.

**Figure 2:**
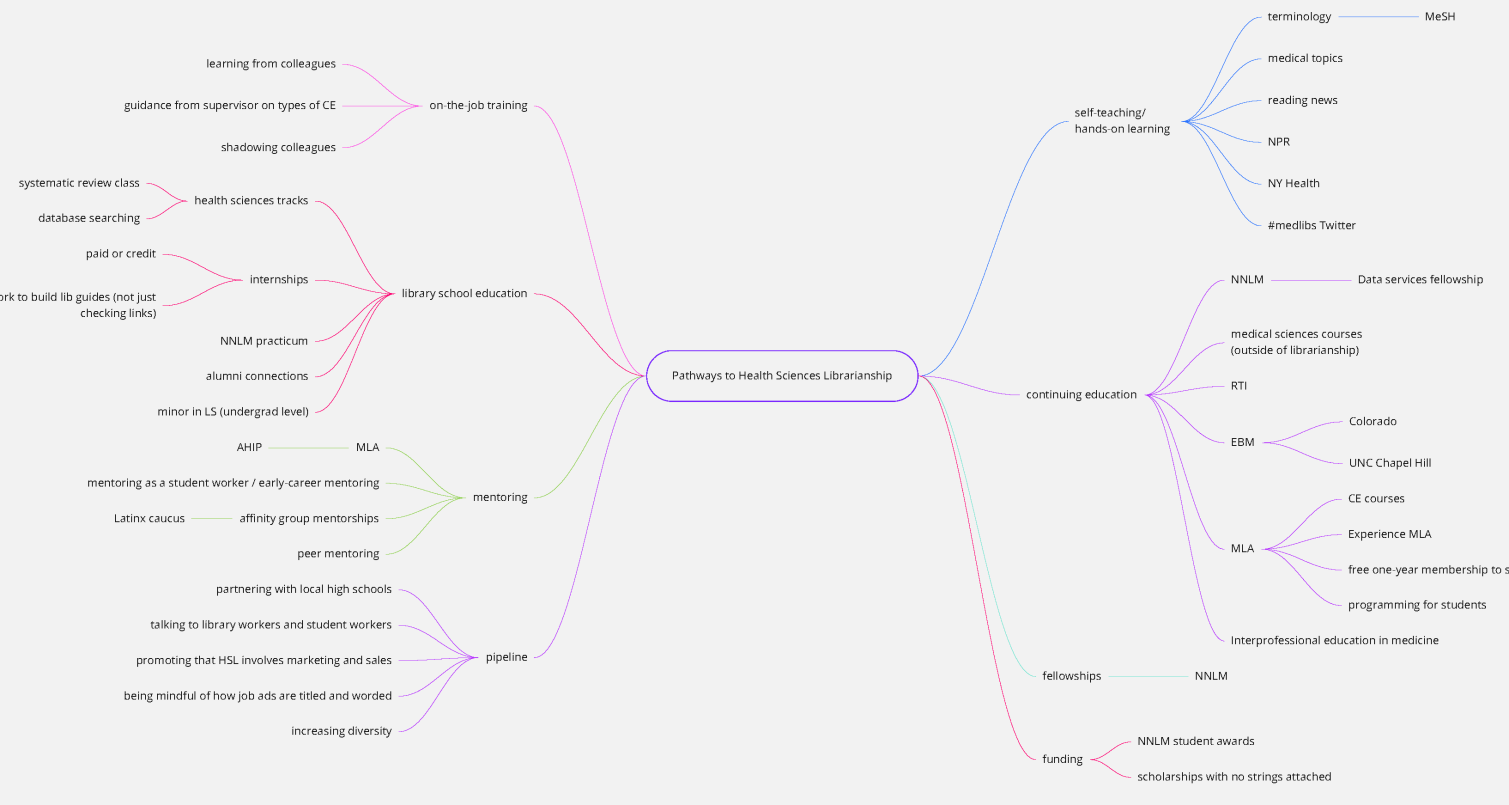
Miro visualization of pathways discussion

As the small groups wrapped up the discussions, facilitators who had been synthesizing the groups' contributions generated a map of themes that had emerged and displayed it on a large screen at the front of the room (figure 2). This image was then presented to the whole group during the last component of the session, providing participants with a summary of their contributions and prompting more discussion.

## RESULTS

The following pathways emerged from the group discussions: library school education; internships and practica; the Library and Information Science (LIS) pipeline; on-the-job training; mentoring; self-teaching/hands-on learning; and continuing education. We share here details about trends and themes regarding each pathway. There is some overlap in the trends and themes, as there was overlap in the discussions.

### Library School Education

**Figure 3:**
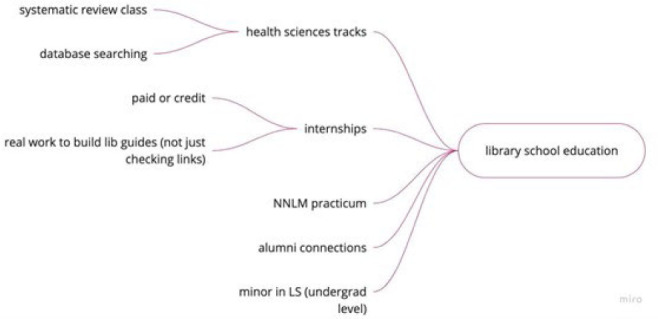
Miro visualization of library school education branch

One of the more formal pathways discussed in the session was library and information science (LIS) graduate programs. However, participants (as outlined in Figure 3) noted that health sciences-focused library school education is not available to everyone. Not all LIS programs have formal health sciences coursework (for a list of library schools with courses in health sciences information refer to the list from the Medical Library Association [6]). There are library schools that do offer health sciences tracks (e.g., health sciences concentrations or certificates). These tracks may include specific courses such as health sciences librarianship or courses that discuss database searching in the health sciences. However, programs that offer courses in health sciences library courses may only offer them sporadically or in limited formats (e.g., only in person or with a restricted number of student seats), underscoring that only a limited number of LIS students are able to focus on health sciences librarianship within an LIS program.

**Figure 4:**
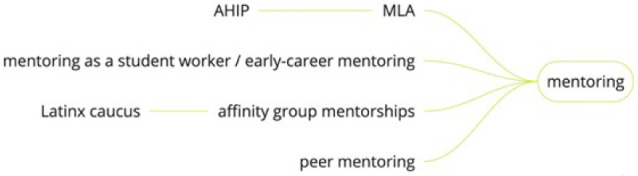
Miro visualization of mentoring branch

**Figure 5:**
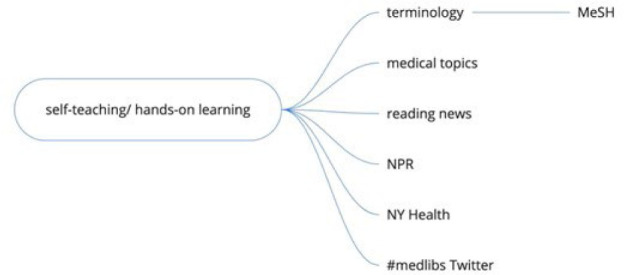
Miro visualization of self-teaching branch

**Figure 6:**
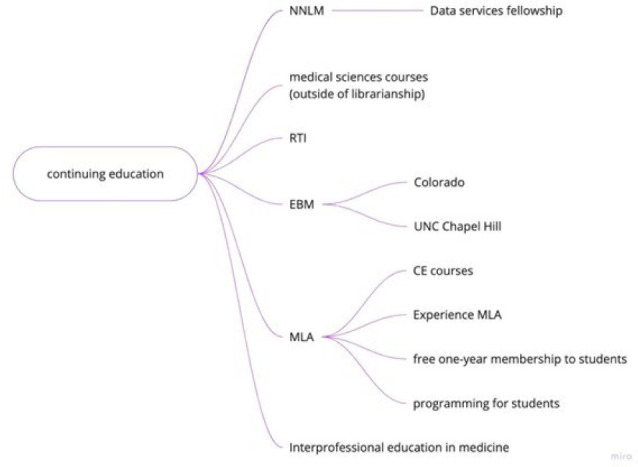
Miro visualization of continuing education branch

**Figure 7:**
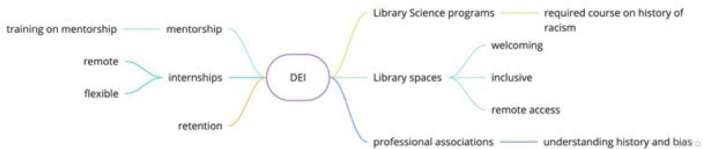
DEI themes emerged across discussions of different pathways

### Internships & Practica

Session participants also noted that outside of formal coursework, library school students may also pursue internships that enable them to explore health sciences librarianship. Internships are a way for LIS students to build their skills and develop professional identities, while also providing an opportunity for current health science librarians to pass on their knowledge and experience. There are a variety of internship types that may be offered for credit or may be paid (e.g., graduate assistantships in health sciences libraries). Immersion session participants noted the importance of internships providing opportunities for library students to do meaningful work such as building LibGuides (i.e., not just checking links on a website) as well as being paid for the internship (rather than libraries relying on unpaid student labor). Participants shared that many internship opportunities are informal and may contain inconsistencies. Moreover, participants noted that for-credit versus paid internships can be a barrier for many LIS students who are working, have caretaking responsibilities, or live in a rural area, themes which have also emerged more generally in the literature on LIS graduate students [7].

In addition, library students may pursue practicum opportunities. Immersion session attendees noted that the Network of the National Library of Medicine (NNLM) offers practica to library students. One example is the NNLM National Center for Data Services, which offered a Data Internship program in Summer 2022 with the goal to provide graduate students from underrepresented racial and ethnic groups with practical experiences and skills for obtaining data librarian positions in health sciences libraries. Additional library school programs designed to develop future health sciences librarians may include alumni connections with practicing health information professions. Session attendees suggested that library schools could promote such opportunities in the health sciences more effectively to their students.

### The LIS Pipeline

Another theme that emerged in the small group discussions was what we have termed the LIS pipeline, or how individuals are introduced to Library and Information Science as a professional field, including professional networking, awareness of job prospects, and policies for librarian retention and promotion. Many LIS leaders and managers are concerned with how to attract and retain qualified library staff and faculty. Within the context of health sciences librarianship, there are specific concerns about how LIS students and professionals learn about - or stumble upon - this specialized field and how knowledge of the field's existence, or lack thereof, impacts diversity, equity, and inclusion in health sciences libraries. Key points that emerged around this theme in the session discussion include demonstrating that a variety of careers and educational backgrounds may produce transferable skills needed for health sciences librarianship and rethinking how job descriptions and advertisements are written for health sciences library positions.

Session participants articulated the need to engage in career outreach early via a variety of avenues. Suggestions included creating outreach opportunities targeted at high school students who may be unfamiliar with health sciences librarianship as a career path as well as offering workshops or shadowing opportunities for LIS students. Health sciences libraries advertising positions may want to carefully consider the position titles in their internship and job descriptions, as some LIS students or current professionals may not apply for the positions with the incorrect assumption that a health sciences background is necessary. Participants also noted issues closely tied to the issue of credit versus paid internships: whether these internship and job descriptions are written with unnecessary library or medical jargon, have flexible hours, or are hybrid or remote [7]. Such concerns about inclusive language and the limitations of rigid schedules and in-person location requirements will likely be important conversations moving forward for many health sciences libraries. Library schools and alumni networks can be vital partners for promoting health sciences librarianship opportunities, which can help expand the pool of prospective candidates for internships, jobs, funding opportunities, etc. MLA and NNLM were seen by immersion session participants as organizations that are conducting outreach and learning opportunities for students through events like Experience MLA and NNLM's trainings, fellowships, and grants. Along with existing programs, attendees suggested that MLA provide free student membership for at least the first year and create more programming for students throughout the year.

Across librarianship there have been tough conversations about whether a master's degree is needed to accurately perform librarian job duties. Session participants noted that many libraries have talented staff and student workers that could be encouraged to move into higher level positions, but the master's degree requirements for many librarian positions can be a hindrance to diverse applicant pools. Some participants expressed interest in considering professionals with different degrees and experiences, while others suggested that librarians and administrators should speak to library staff and student workers about pursuing specialized librarian pathways. Personal mentorship could encourage student and library workers from underrepresented backgrounds and experiences to pursue advanced higher education.

Attendees shared concerns about how job descriptions and advertisements for health sciences library positions and internships are written in ways that create unnecessary barriers for entry into the field. Some participants explained that human resources and institutional bureaucracy can make changing job descriptions difficult, thus impairing a library's ability to update positions or alter language to be more inclusive. Regarding accessibility, it was suggested that for some positions it is not necessary to include outdated language related to lifting or carrying heavy items, while others shared that they thought medical jargon could be intimidating. Additionally, participants suggested that libraries should be more conscious about where they are sharing and advertising their new positions; specifically sharing positions with library schools and affinity groups may help reach more applicants.

Participants discussed additional barriers for diversity in health sciences library hiring practices. For example, participants expressed concerns about recruiting diverse hires when institutions may be unwelcoming environments with limited opportunity for advancement. Health sciences libraries have inclusion and diversity challenges that simply disseminating more information about opportunities alone will not fix.

### On-the-Job Training

Another pathway mentioned in the small group discussions was on-the-job training. Specifically, participants mentioned learning from colleagues and shadowing them, as well as seeking guidance from supervisors about continuing education courses to pursue. Related to these examples that emerged in the discussions are examples in the literature about guiding early career librarians in seeking on-the-job training opportunities as well as supervisors onboarding new health sciences librarians.

Bartley, Simuel, and Williams have published tips for librarians new to health sciences, including a list of questions a librarian can ask themselves, their director, or supervisor to learn more about the institution and possible opportunities for professional development [2]. Health sciences librarians use a variety of methods to learn onthe-job. For example, librarians viewing each other's literature searches can provide ongoing training through observation and feedback [8]. In regards to librarians teaching in the health sciences, case studies highlight peer observation as an essential component of learning about teaching styles and session structures [9]. Beyond observing immediate colleagues within the library, librarians often observe and collaborate with clinicians and epidemiologists when developing evidence-based practice classes [10]. Library managers can take advantage of Structured On-the-Job Training models developed in the field of human resource development to be more intentional in preparing new health sciences librarians, presenting specific training, providing feedback, and evaluating the performance of the library trainee [11]. Structured on-the-job training may benefit new health sciences library hires, while ensuring consistency in the services offered by the library.

### Mentoring

Participants in the session frequently mentioned the importance of mentoring as a pathway to health sciences librarianship. Participants shared a variety of formal and informal mentoring experiences that they had been involved in as mentors and/or mentees. Formal mentoring experiences include the mentoring that is part of the process for applying for MLA's Academy of Health Information Professionals (AHIP) credential, in which applicants for the credential are paired with a mentor who guides them through the process. MLA affinity caucuses, such as the Latinx Caucus and the African American Medical Librarians Alliance (AAMLA), also sponsor mentoring programs for early-career librarians in their communities. MLA, the American Library Association, and the National Library of Medicine sponsor the Spectrum Scholarship, which connects BIPOC library students and early-career librarians with mentors and job shadowing experiences [12]. Other mentoring programs include a cohort of LIS students and early-career librarians interested in health sciences librarianship who were sponsored by a grant from the Network of the National Library of Medicine to take a 4-week, online Introduction to Health Sciences Librarianship course while being paired with an experience health sciences librarian mentor [3].

In addition to mentoring programs sponsored by professional organizations, session participants also mentioned how libraries may have their own mentoring programs. Such programs include mentoring programs for student workers, interns, or early-career librarians. Some libraries also integrate mentoring into on-the-job training, such as peer mentoring programs, where health sciences librarians mentor each other through practices such as shadowing research consultations or instruction sessions, skill sharing, and peer feedback. Session participants also mentioned finding mentors, or mentoring others, outside of formal mentoring programs. An idea that emerged in one of the discussions was for a peer mentoring program that could match pairs of librarians who share the experience of transitioning to health sciences librarianship from other library backgrounds or other careers.

### Self-teaching/Hands-on Learning

Another theme that emerged from the discussion groups was the use of self-teaching and hands-on learning to develop the skills needed to work as a health sciences librarian. Participants mentioned approaches to learning the unique clinical and biomedical terminology encountered working in a health sciences library, especially the importance of reviewing Medical Subject Headings (MeSH) to learn medical terminology. Participants also mentioned using self-teaching to develop more familiarity with medical topics, such as following news on medical topics in sources such as National Public Radio and the New York Times Health section. There was also mention of following the social media accounts of health organizations such as Centers for Disease Control and Prevention and World Health Organization.

Participants also described methods they used to teach themselves specifically about health sciences librarianship. Many participants monitored #medlibs on Twitter. Some participants asked other health sciences librarians questions on Twitter to learn about health sciences librarianship and stay up to date with the field. Attendees also described spending time teaching themselves the basics of PubMed because they felt it was the most important database for health sciences librarians to know and is freely available.

### Continuing Education

The common experience of finding oneself in a health sciences librarian position and not having acquired in library school a health sciences skill set or any prior training in the health sciences led to a discussion of continuing education opportunities among participants. While some continuing education opportunities are freely available, support for fee-based continuing education may be available from the institution providing the education or from one's institutional professional development funds, MLA, a local MLA chapter, or a NNLM regional library.

Continuing education programs mentioned during the session include: data services training, health sciences courses (non-library), MLA's Research Training Institute, evidence-based practice courses, bioinformatics, reference training, consumer health, multilingual & multicultural health information, drug information & drug terminologies, as well as other CE course offerings. MLA provides a list of professional competencies in health sciences librarianship [16]. As a reference, Appendix I lists CE opportunities mentioned during the immersion session as well as additional ones that new health sciences librarians may find useful but is not an exhaustive list.

### Pathways for Equity, Diversity, and Inclusion

Themes related to diversity, equity, and inclusion emerged throughout the immersion session discussions – particularly issues of recruitment, retention, and pay. The format of the session allowed topics to emerge that might not have emerged in a formal survey. The whole group discussion after the small group discussions ended up focusing on DEI questions. Questions that emerged include: Who sees paths to health sciences librarianship available to them? Who stays in the profession and who leaves? How does diverse librarian retention relate to future recruitment? Such issues relate to work currently being done in MLA affinity caucuses.

To make LIS programs more equitable, diverse, and inclusive, some session participants suggested a required course on the history of racism in libraries as part of the LIS curriculum. To build pathways to health sciences librarianship that can create a more diverse, equitable, and inclusive profession, the kind of community-building fostered in the immersion session will need to further include wider participation and not be limited to those able to participate in-person at an MLA conference.

## DISCUSSION

Because there are multiple pathways to health sciences librarianship, making more visible the ways that someone may approach entering this area of librarianship can have implications on who becomes a health sciences librarian and how the profession retains librarians. Making pathways more visible can help remove unnecessary and inequitable barriers to entry in the profession and thus strengthen the field of health sciences librarianship. That there are different paths to health sciences librarianship does not have to be “hidden knowledge.” There can be more public knowledge about pathways for LIS students and others considering applying to health sciences library positions.

Participants in the immersion session did not typically describe a linear path into the profession. Instead, participants tended to describe how they developed needed skills by cobbling together learning opportunities and professional development. The comments collected in Jamboard and visualized thematically in Miro provide a map of paths for building the expertise needed for a career in health sciences libraries. A variety of themes emerged in each category. Some of these themes were suggestions on how to improve a particular pathway. Other themes simply revealed a method being used for learning. Additionally, the discussions called attention to possibilities for improving and better promoting existing programs and developing new programs to address unmet needs. Many of these themes are interconnected and circle back to the importance of diversity, equity, and inclusion in health sciences librarianship.

LIS programs can help establish a more formal pathway to health sciences librarianship. This formal pathway could include health sciences librarianship skills-based courses on topics such as systematic reviews and database searching. LIS programs could also offer paid health sciences library internships and practica that allow students to get practical, hands-on experience. Such additions to LIS programs could enhance LIS students' ability to obtain a health sciences librarian position quickly after graduation and succeed in it. Establishing a more formal pathway and one that offers paid internships and practica could also positively impact recruitment and retention of diverse applicants in health sciences librarianship.

Health sciences libraries can help improve the diversity, talent, and quality of the health sciences librarian applicant pool by being intentional about position descriptions. For example, if it is not necessary for a candidate to have health sciences experience, the job description should specify that. An additional way to improve the LIS pipeline is to develop opportunities for library staff, undergraduate, and high school students to learn more about health sciences librarianship.

Although it is common to learn from peers in health sciences libraries, library managers could take advantage of structured on-the-job training models. This would provide a more intentional model including specific training, feedback, and performance evaluation of the new health sciences librarian. Closely tied to on-the-job training, formal and informal mentoring programs are important, including mentoring programs organized within a library or through professional organizations. New mentoring programs could be developed, such as a program that would pair librarians with the shared experience of transitioning to health sciences librarianship from other library backgrounds or other careers.

Self-teaching and hands-on-learning may utilize a mix of resources created specifically for health sciences information, such as free NNLM online training on MeSH and PubMed, as well as more general resources, such as news sources on medical topics. Health sciences community-created resources, such as Twitter posts and Discord conversations, are also important because they help answer practical questions and create community for a new professional. Curiously, participants in the immersion session did not mention staying current with new professional publications. However other case studies have noted self-teaching in the form of reading blogs and books, most frequently The Accidental Health Sciences Librarian [13], as an important supplement to formal health sciences librarianship training [9]. Other studies have shown that reading medical journal articles was one form of self-teaching for librarians teaching in evidence-based medicine curricula [10].

There are a vast array of CE opportunities including free, low cost, and paid options. Development of any new CE opportunities should begin with a review of MLA competencies [16] so that resources can best be allocated. CE is a way to gain knowledge, expand skill sets, and enrich the work being performed. There are however many barriers to accessing CEs, particularly financial and time commitment issues. It is important to acknowledge how common on-the job continuing education, self-teaching, mentoring, and hands-on learning are in the field. Otherwise, diverse applicants may be less likely to apply for health sciences librarian jobs because they will assume that they need knowledge of the health sciences and/or health sciences librarianship prior to starting a position. It could help for job ads for health sciences librarian positions to transparently say if health sciences knowledge is required, preferred, or could be learned onthe-job.

## LIMITATIONS

There are limitations to the way the pathways outlined in this article were identified. The findings presented in this article were derived from in-person participation in the Medical Library Association conference in New Orleans; there is a limitation inherent to who can attend an in-person meeting with a registration fee. The voices of those who were not able to attend the in-person meeting are not included in this discussion; there are thus limitations in the demographics of those who could contribute to the discussion. Participation was by a self-selecting group of approximately 30-45 people who selected to attend the immersion session.

The method of having people participate in small groups with facilitators thematically analyzing discussions using the Miro visualization tool was effective for picking up themes. However, it was less effective for picking up all the examples mentioned, as it was difficult to enter in all the details in real time. Thus, future iterations of such interactive, immersive sessions may want to consider having both a facilitator and note-recorder in each small group.

## CONCLUSION

The immersion session described in this article models an approach to building professional community, by collaboratively mapping the many pathways that librarians take to entering and developing careers in health sciences librarianship. When knowledge about how to enter and succeed in the library profession is not visible and widely disseminated, there is a particular impact on library workers from marginalized communities [17]. Librarians may thus want to consider conducting similar exercises as this immersion session at the local level. While it is valuable to create opportunities for discussion, it is even more important to publicly share the findings of these discussions and make pathways to health sciences librarianship more visible.

This article also hopes to serve as a model for publicly reflecting on and sharing conference session findings, by utilizing open access publication to make public the knowledge generated in a fee-based conference. We aimed to map and uncover knowledge about pathways to health sciences librarianship, documenting how librarians arrive in the niche field of health sciences librarianship, and examining what might be causing the lack of diversity and talent that we hope for in our profession. Future steps should include mapping out barriers to specific pathways to health sciences librarianship, to directly address barriers and not only discuss them.

## SUPPLEMENTAL FILES

**Appendix A:** Continuing Education Opportunities
