# Supplementary material for: Mapping the pathways to health sciences librarianship: reflections and future implications from an immersion session
Source: J Med Libr Assoc. 2023 Oct 2;111(4):802–10. doi: 10.5195/jmla.2023.1645 (PMC10621722; doi:10.5195/jmla.2023.1645)
Supplement: Supplementary file 1 — Appendix A: Continuing Education Opportunities [file jmla-111-4-802-s01.pdf]

Appendix A: Continuing Education Opportunities

**Type of CE:**	**Example Offerings:**
Data Science	NNLM's data services fellowship (https://nnlm.gov/guides/data-services)<br><br>NLM RDM On-demand: Data Sharing and Publishing, https://nnlm.gov/training/class/rdm-demand-data-sharing-and-publishing<br><br>NNLM Fundamentals of HS Research Data Management, https://nnlm.gov/training/class-catalog/fundamentals-health-sciences-research-data-management
Data Science-Bioinformatics	NLM Diversity in Data Science and Informatics (DDSI) Research Summer Internship Program, https://www.nlm.nih.gov/research/DDSI.html<br><br>NLM A Librarian's Guide to NCBI, https://www.ncbi.nlm.nih.gov/education/librarian/<br><br>NNLM Bioinformatics and Biology Essentials for Librarians: Databases, Tools, and Clinical Applications, https://nnlm.gov/training/class-catalog/bioinformatics-and-biology-essentials-librarians-databases-tools-and
Research Data Outreach Award	NNLM, https://nnlm.gov/funding/rfa/research-and-data-outreach-award
Research (want to conduct a research project with help from a mentor and guidance)	MLA's Research Training Institute (RTI, https://www.mlanet.org/rti)
Public Health	CDC Introduction to Public Health, https://www.cdc.gov/training/publichealth101/public-health.html
Medical Terminology	https://www.rasmussen.edu/degrees/health-sciences/blog/basic-medical-terms/

	https://www.dmu.edu/medterms/
Evidence Based Practice	University of North Carolina Chapel Hill LIS course, https://sils.unc.edu/programs/ebm<br><br>Strauss Health Sciences Library, University of Colorado Anschutz Medical campus EBCP for Librarians Institute, https://library-cuanschutz.libguides.com/ebpml<br><br>University of Chicago at Illinois, https://researchguides.uic.edu/ebm
NLM Associate Fellowship Program	https://news.nnlm.gov/region_4/accepting-applications-for-the-2022-nlm-associate-fellowship-program/
Interprofessional Education	https://www.aamc.org/what-we-do/mission-areas/medical-education/interprofessional-education<br><br>https://www.mededportal.org/interprofessional-education<br><br>Article on health sciences librarians and nursing faculty developing a course, https://onlinelibrary.wiley.com/doi/full/10.1111/hir.12184
Reference Skills for Nursing and Allied Health Research	NNLM offering, https://nnlm.gov/training/class-catalog/building-reference-skills-nursing-and-allied-health-research
PubMed Essentials	NNLM, https://nnlm.gov/training/class-catalog/pubmed-essentials-demand
Experience MLA (free one month membership to learn about MLA)	https://www.mlanet.org/p/cm/ld/fid=1815
MLA Student Membership rates	At the time of writing this article, $61/year (75% off), https://www.mlanet.org/join
MLA Student Programming	MLA caucuses including the New Members Caucus and the Medical Library Education Caucus offer programming for LIS students. Experience MLA (see above in the table) is an annual event that also offers programming that may be of interest to students.

To find additional CE offerings	MLA CE offerings, MED-LIB ED, http://www.medlib-ed.org/
	NNLM offerings, https://nnlm.gov/training
	Library Juice Academy https://libraryjuiceacademy.com/
